# MRD positivity was the poor prognostic factor for adverse-risk AML patients with allogeneic hematopoietic stem cell transplantation: a multicenter TROPHY study

**DOI:** 10.1038/s41408-024-00976-1

**Published:** 2024-01-17

**Authors:** Yang Cao, Wenxuan Huo, Jiayu Huang, Yang Yang, Yu Wang, Yingjun Chang, Luxiang Wang, Zilu Zhang, Chuanhe Jiang, Xiaoxia Hu, Xiaodong Mo

**Affiliations:** 1 Department of Hematology, Tongji Hospital, Tongji Medical College, Huazhong University of Science and Technology, Wuhan, Hubei 430030 China; 2 Peking University People’s Hospital, Peking University Institute of Hematology, National Clinical Research Center for Hematologic Disease, Beijing Key Laboratory of Hematopoietic Stem Cell Transplantation, Beijing, 100044 China; 3 Research Unit of Key Technique for Diagnosis and Treatments of Hematologic Malignancies, Chinese Academy of Medical Sciences (2019RU029), Beijing, 100044 China; 4 State Key Laboratory of Medical Genomics, Shanghai Institute of Hematology, National Research Center for Translational Medicine, Shanghai Rui Jin Hospital, Shanghai Jiao Tong University School of Medicine, Shanghai, 200025 China; 5 Collaborative Innovation Center of Hematology, Shanghai Jiao Tong University School of Medicine, Shanghai, 200025 China

**Keywords:** Acute myeloid leukaemia, Acute myeloid leukaemia

Dear editor,

Acute myeloid leukemia (AML) is a highly heterogeneous disease distinguished by different cytogenetic and genetic characteristics [1, 2]. Currently, the risk classification based on the cytogenetics and molecular markers (e.g., the European LeukemiaNet [ELN] risk stratification) is the mainstay criterion that direct the treatments of adult AML patients, and those with adverse-risk AML are recommended to receive allogeneic hematopoietic stem cell transplantation (allo-HSCT) in their first complete remission (CR1) [1, 3]. Several studies have reported that the efficacy of allo-HSCT is superior to that of those receiving consolidation chemotherapy alone in adverse-risk AML patients and the benefit of allo-HSCT is observed across ages and donor type [4].

Measurable residual disease (MRD) detected by multiparameter flow cytometry (MFC) is the commonly used approach to predict post-transplant relapse in AML [5–9]. Many studies reported that the risk of post-transplant relapse significantly increased in patients who were MFC positivity before allo-HSCT [10–12]; however, some authors suggested that pre-transplant MFC MRD was less important in predicting relapse than variables reflecting the biology of the disease (e.g., cytogenetics) [13]. Thus far, the prognostic value of pre-HSCT MFC MRD positivity is still controversial in AML patients. In addition, no study had compared the clinical outcomes between patients who were MRD positivity and MRD negativity in the adverse-risk AML group. In the present study, we aimed to identify the prognostic value of pre-HSCT MFC MRD positivity in patients with adverse-risk AML, which may further optimize the timing of allo-HSCT.

This multicenter, retrospective study based on the transplant database of Wuhan Tongji Hospital, Shanghai Ruijin Hospital, and Peking University Institute of Hematology (PUIH) (i.e., TROPHY group). Consecutive AML patients receiving allo-HSCT from January 2017 to June 2022 were screened, and the eligibility criteria were as follows: (1) aged ≥ 16 years; (2) adverse-risk AML based on ELN 2022 criteria; (3) achieving CR1 before allo-HSCT. The last follow-up was June 30, 2023. The study was approved by the institutional review board of each participated hospital and was conducted in accordance with the *Declaration of Helsinki*.

The protocols for preconditioning regimen, graft-versus-host disease prophylaxis, and infection prophylaxis were reported previously [14, 15]. MRD status was monitored after consolidation chemotherapy, before allo-HSCT and at 1, 2, 3, 4, 5, 6, 9, and 12 months after allo-HSCT and at 6-month intervals thereafter. Leukemia-associated aberrant immunophenotypes (LAIPs) and/or different from normal (DfN) is identified by MFC (Supplementary method) and 0.1% was applied as a threshold to distinguish MRD-positivity. MRD-negative patients received therapy for relapse prophylaxis after allo-HSCT was defined as maintenance therapy. For patient who were MRD positivity or MRD reoccurrence after allo-HSCT, they received preemptive therapy, such as donor lymphocyte infusion (DLI) and interferon-α (Supplementary method).

Data were censored at the time of death or last available follow-up. The primary outcome was relapse. The secondary outcomes included non-relapse mortality (NRM), event-free survival (EFS), leukemia-free survival (LFS), and overall survival (OS) (Supplementary method). Frequency and percentage were used to describe the characteristics of patients. The Kaplan–Meier estimator was used to calculate the probabilities of survival, and the cumulative incidence function was used to calculate the incidence of relapse and NRM with competing risk analysis. Additionally, landmark analyses were performed to assess outcomes within one year and between 1 year and 2 years after allo-HSCT. Two-sided *P*-values were adopted. The univariable and multivariable Cox regression was performed to determine the impact of potential prognostic factors on clinical outcomes (Supplementary method). Independent variables with *P* > 0.1 were sequentially excluded from the model, and *P* < 0.05 was considered to be statistically significant. The association between MRD and endpoints (relapse and death) were evaluated on a log10-transformed continuous variable with restricted cubic spline curves based on logistic regression model. Statistical analysis was performed using the R software 4.2.0 (https://www.r-project.org) and Statistical Package for the Social Sciences 26 (SPSS Inc., IBM, Armonk, NY, USA).

A total of 391 adverse-risk AML patients were enrolled, and the characteristics were showed in Supplemental Table 1. The median follow-up was 759 days (range: 707–811 days). Fifty-two patients experienced relapse, and 36 patients died of NRM. The information of GVHD was summarized in Supplementary Table 2. The 2-year probability of relapse, NRM, LFS, and OS after allo-HSCT was 14.9% (95% CI: 11.0%–18.8%), 10.0% (95%CI: 6.9%–13.2%), 74.6% (95%CI: 70.0%–79.5%), and 83.8% (95%CI: 80.0%–87.8%), respectively.

**Fig. 1:**
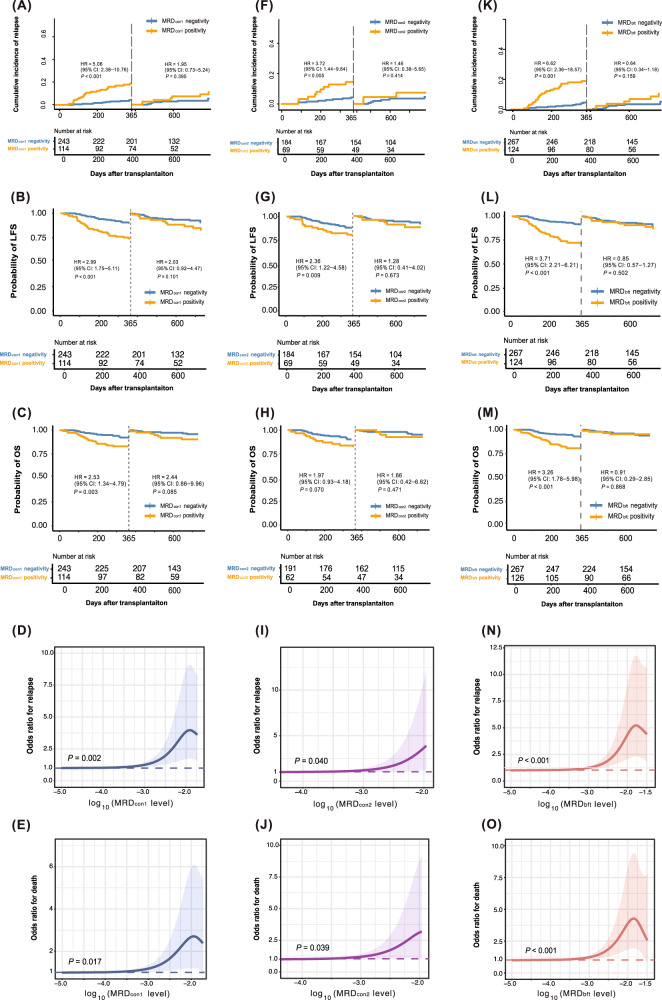
The 2-year probabilities of clinical outcomes according to MRD status for patients with adverse-risk AML receiving allo-HSCT. (**A**) relapse, (**B**) LFS and (**C**) OS after the first consolidation chemotherapy; the effect of MRD_con1_ level on (**D**) relapse and (**E**) death; (**F**) relapse, (**G**) LFS and (**H**) OS after the second consolidation chemotherapy; the effect of MRD_con2_ level on (**I**) relapse and (**J**) death; (**K**) relapse, (**L**) LFS and (**M**) OS before transplantation; the effect of MRD_bft_ level on **(N**) relapse and (**O**) death.

**Fig. 2:**
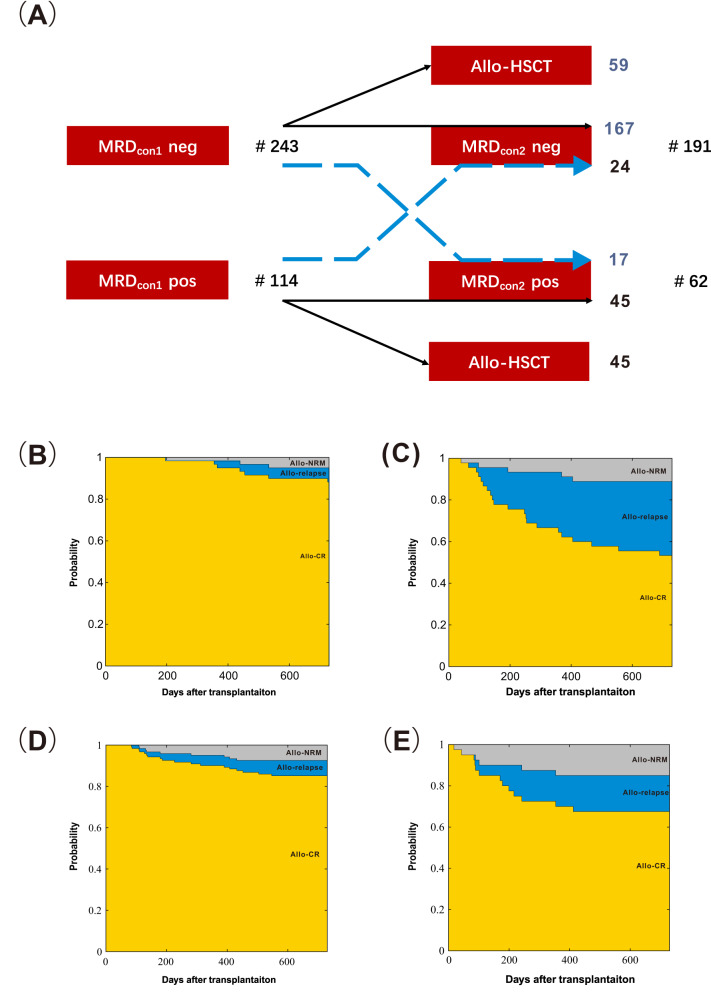
The change of MRD status after the first consolidation chemotherapy and clinical outcomes according to MRD status after the first and the second consolidation chemotherapy. (**A**) Description and transition of MRD status after the first consolidation chemotherapy. Probabilities of clinical outcomes for patients with MRD_con1_ negativity (**B**) and with MRD_con1_ positivity (**C**) receiving allo-HSCT after the first consolidation directly; Probabilities of clinical outcomes for patients with MRD_con2_ negativity (**D**) and with MRD_con2_ positivity (**E**) receiving allo-HSCT after the second consolidation directly.

We firstly analyzed the influence of MRD status after the first consolidation chemotherapy (MRD_con1_) on post-transplant outcomes. A total of 114 patients showed MRD_con1_ positivity (Supplementary Fig. 1). Among the patients with MRD_con1_ positivity, 87 (76.3%) of them achieved MRD negativity after allo-HSCT. The probabilities of relapse, EFS, LFS, and OS at 2 years after allo-HSCT were all superior in MRD_con1_ negative group compared with MRD_con1_ positive group, and the benefit of MRD_con1_ negativity was more pronounced within the first year after transplantation (Fig. 1, Supplementary Table 3). Similar results were observed in the 104 patients receiving allo-HSCT directly after the first cycle of consolidation chemotherapy (MRD_con1_ positivity: *n* = 45; MRD_con1_ negativity: *n* = 59) (Fig. 2B, C, Supplementary Table 4). In multivariable analysis, MRD_con1_ positivity was associated with a poorer LFS after being adjusted by other variables (Supplementary Fig. 2, Supplementary Table 5).

We secondly analyzed the prognostic value of MRD status after the second consolidation chemotherapy (MRD_con2_) on post-transplant outcomes. For 253 patients received two cycles of consolidation chemotherapies, 62 (24.5%) showed MRD_con2_ positivity (Supplementary Fig. 1). Particularly, among patients with MRD_con1_ positivity (*n* = 69), 24 (34.8%) turned MRD negativity after the second round of consolidation (Fig. 2A). 51 of 69 (82.3%) patients with MRD_con2_ achieved MRD negativity after allo-HSCT. The probabilities of relapse, EFS, and LFS of MRD_con2_ negative group at 2 years after allo-HSCT were all superior as compared with those of MRD_con2_ positive group (Fig. 1, Supplementary Table 6). Similarly, for the 161 patients receiving allo-HSCT directly after the second cycle of consolidation chemotherapy (MRD_con2_ positivity: *n* = 40; MRD_con2_ negativity: *n* = 121), MRD_con2_ negativity was associated with better clinical outcomes. (Fig. 2D, E, Supplementary Table 7). Of note, the clinical outcomes were comparable between patients who were MRD_con1_ negativity and MRD_con2_ negativity before allo-HSCT (Supplementary Table 8). Multivariable analyses identified MRD_con2_ positivity was an independent adverse prognostic factor for relapse after being adjusted by other variables (Supplementary Fig. 2, Supplementary Table 5).

We further analyzed the impact of MRD status before transplantation (MRD_bft_) on post-transplant outcomes. A total of 124 patients showed MRD_bft_ positivity (Supplementary Fig. 1). 97 (78.2%) patients achieved MRD negativity after allo-HSCT. The probabilities of relapse, EFS, LFS, and OS at 2 years after allo-HSCT were all superior in MRD_bft_-negative group compared with MRD_bft_-positive group, particularly within the first year after allo-HSCT (Fig. 1, Supplementary Table 9). In multivariable analysis, the MRD_bft_ positivity was associated with a poorer EFS after being adjusted by others variables (Supplementary Fig. 2, Supplementary Table 5).

The maintenance and preemptive therapy after allo-HSCT were lastly analyzed. 344 patients achieved MRD negativity after allo-HSCT, and 60 (17.4%) of them received maintenance therapy (hypomethylating agents [HMA]: *n* = 50; tyrosine kinase inhibitors [TKI]: *n* = 10), and the median time from allo-HSCT to initiation of maintenance therapy was 108 days (range 13–511). Fifty-one patients who showed MRD positivity after allo-HSCT received preemptive therapies (DLI: *n* = 23; IFN-α: *n* = 38). MRD_bft_-positive patients receiving maintenance therapies had a better OS compared with those without maintenance therapies (Supplementary Table 10-11). In addition, although MRD_bft_-negative patients without maintenance therapies had a superior EFS and LFS compared with MRD_bft_-positive patients receiving maintenance therapies, the 2-year probability of OS did not differ between the two groups (Supplementary Table 12). The clinical outcomes of patients receiving preemptive therapies were showed in Supplementary Table 13.

Some studies reported that MFC status before allo-HSCT could not predict relapse after allo-HSCT [16–20], nevertheless, other factors, such as chemotherapy resistance [18], disease status beyond CR1 [16, 18], or adverse cytogenetics [17] are independent risk factors for post-HSCT relapse. Thus, some investigators suggested that factors reflecting underlying disease biology may be more important for predicting relapse compared with MFC positivity [13]. However, there were some limitations for these studies, for example, the MRD was performed by 4-color MFC analysis [19], the cut-off values for MFC positivity were relatively low (0.001%-0.01%) [16–18], or the ratios of patients with adverse-risk AML were low (~10%) [12, 16]. We firstly identified the prognostic value of MFC MRD detected at three critical timepoints before allo-HSCT in a disease-specific population of adults with adverse-risk AML, and our results provided a valuable experience for exploring the up-to-date undefined role of pre-HSCT MFC status in these patients.

We observed that for those who were MRD_con1_ positivity and receive allo-HSCT straightly, the relapse rate could be as high as 42%. Nearly one third of them could achieve MRD_con2_ negativity after the second consolidation chemotherapy and the relapse rate after allo-HSCT was only 8% in MRD_con2_-negative patients. Although the other two-thirds of patients could not achieve MRD_con2_ negativity, the relapse rate of patients who were MRD_con2_ positivity and receiving allo-HSCT after the second consolidation chemotherapy was only 17.6%. Therefore, we suggested that an effective second consolidation chemotherapy may help to further deepen the response of treatment and decrease the relapse risk. But on the other hand, considering that only a minority converted to MRD negativity after the second consolidation chemotherapy, patients might also consider receive allo-HSCT after the first consolidation regardless of MRD status. This also suggested that how to achieve MRD_con2_ negativity with new drugs or novel therapeutic protocols might be critical to further improve the clinical outcomes of MRD_con1_-positive patients.

On the contrary, we observed that nearly 90% of patients who were MRD_con1_ negativity reserved MRD negativity after the second consolidation (i.e., MRD_con2_ negativity). Considering that the relapse rate was 11% and 8.2% for MRD-negative patients who proceeded to allo-HSCT directly after the first and second course of consolidation, respectively, and additional courses of consolidation chemotherapies might increase the risk of organ toxicities or infection, the patients with MRD_con1_ negativity seemed to benefit less from the second consolidation. Thus, adverse-risk AML patients who achieved MRD_con1_ negativity might receive allo-HSCT directly after the first consolidation chemotherapy without further consolidation.

Many studies had identified the efficacy of maintenance therapy in high-risk AML patients [21–23], but the results were somewhat controversial. For example, some reported that sorafenib maintenance can decrease relapse and improve LFS after allo-HSCT [24]; however, MORPHO trial reported that relapse-free survival and OS were comparable between patients with and without gilteritinib maintenance, and gilteritinib maintenance might only improve the survival of patients who were MRD positivity before allo-HSCT [25]. In our study, we also observed that only the MRD_bft_-positive patient may benefit from maintenance therapies. This may help to further recognize the adverse-risk AML patients who might truly benefit from maintenance therapy after allo-HSCT.

In conclusion, this was the largest study identifying the prognostic value of MRD positivity in adverse-risk AML patients who receiving allo-HSCT in CR1. Patients who achieved MRD_con1_ negativity could benefit more from allo-HSCT directly and those with MRD_bft_ positivity may benefit more from maintenance therapy. Our results could be further confirmed by multicenter randomized controlled trials in the future.

### Supplementary information

Supplementary material
