# Supplementary material for: MRD positivity was the poor prognostic factor for adverse-risk AML patients with allogeneic hematopoietic stem cell transplantation: a multicenter TROPHY study
Source: Blood Cancer J. 2024 Jan 17;14(1):8. doi: 10.1038/s41408-024-00976-1 (PMC10791633; doi:10.1038/s41408-024-00976-1)
Supplement: Supplementary file 1 — Supplementary material [file 41408_2024_976_MOESM1_ESM.docx]

**Supplemental Appendix**

**MRD positivity was the poor prognostic factor for adverse-risk AML patients with allogeneic hematopoietic stem cell transplantation: a multicenter TROPHY study**

**Contents**

**Supplementary Methods**

MFC detection of MRD 2

Protocol for preemptive donor lymphocyte infusion 2

Protocol for preemptive interferon-α treatment 2

Definitions of endpoints 2

Variables included in univariable and multivariable analysis 2

**Supplementary Figures**

Supplementary Figure 1 Diagram of patients enrolled. 4

Supplementary Figure 2. Multivariable analysis of risk factors for clinical outcomes after allo-HSCT 5

**Supplementary Tables**

Supplementary Table 1 Characteristics of adverse-risk AML patients receiving allo-HSCT 6

Supplementary Table 2 GVHD after allo-HSCT 7

Supplementary Table 3 Clinical outcomes in patients with positive and negative MRD_con1_ 8

Supplementary Table 4 Clinical outcomes in patients receiving allo-HSCT directly after the first consolidation chemotherapy 9

Supplementary Table 5 Univariable analysis of clinical outcomes following allo-HSCT 10

Supplementary Table 6 Clinical outcomes between patients with positive and negative MRD_con2_

16

Supplementary Table 7 Clinical outcomes in patients receiving allo-HSCT directly after the second consolidation chemotherapy 17

Supplementary Table 8 Clinical outcomes between patients receiving allo-HSCT directly with negative MRD_con1_ and negative MRD_con2_ 18

Supplementary Table 9 Clinical outcomes between patients with positive and negative MRD_bft_. 19

Supplementary Table 10 Clinical outcomes in patients with and without maintenance chemotherapy after allo-HSCT 20

Supplementary Table 11 Clinical outcomes in MRD_bft_ positive patients with and without maintenance therapy 21

Supplementary Table 12 Clinical outcomes between MRD_bft_ positive patients receiving maintenance therapies and MRD_bft_ negative patients without receiving maintenance therapies 22

Supplementary Table 13 Clinical outcomes between patients receiving maintenance and preemptive therapies after allo-HSCT 23

**Supplementary methods**

**MFC detection of MRD**

Eight-color MFC was performed in all patients as a routine clinical test on bone marrow aspirate samples that were obtained as part of baseline assessment before and after HSCT according to previous studies.^1-4^ A panel of eight antibody combinations that recognize CD7, CD11b, CD13, CD14, CD16, CD19, CD33, CD34, CD38, CD41, CD45, CD56, CD61, CD64, CD71, CD117, CD123, and HLA-DR was used for MRD detection, and 0.2–1 million events per tube were acquired on a FACS Cant II. The isotype control monoclonal antibodies were used. Positive MRD was considered when a cluster of more than 25 cells with leukemia-associated immunophenotypes (LAIP) and SSC characteristics identified in all plots of interest and carrying at least two LAIP markers identified at diagnosis was observed. For those without LAIP markers at diagnosis, MRD was identified as a cell population showing deviation from the normal patterns of antigen expression seen on specific cell lineages at specific stages of maturation compared with either normal or regenerating marrow.^5^ A lower limit of detection (LOD) of 0.01% was targeted. When abnormal cells were identified, the cells were quantified as a percentage of the total CD45+ white cell events. Any measurable level of MRD was considered positive. The standardized assays and quality controls were performed according to previous reports.^6, 7^

**Protocol for preemptive donor lymphocyte infusion**

Granulocyte colony-stimulating factor mobilized peripheral blood stem cells were administered instead of the more common unstimulated donor blood lymphocytes. The initial dose of mononucleated cells (MNCs) for DLI and the dose of cells for repetitive infusion were 1-2×10^8^ MNCs/kg. DLI doses were also defined as CD3^+^ cells per kilogram of recipient weight (1.0× 10^7^/kg -10.0 × 10^7^/kg). Patients could also receive anti-leukemic chemotherapy 48–72 hours before DLI. Patients received immunosuppressive drugs such as ciclosporin A (CSA) to prevent GVHD after DLI for 6–8 weeks at the discretion of the attending physicians (and usually depending on the patient’s GVHD status after DLI). The starting dosage of CSA was 2.5 mg·kg^−1^·day^−1^, which was adjusted to maintain a plasma concentration > 100 ng/mL.^8, 9^

**Protocol for preemptive interferon-α treatment**

Recombinant human IFN-α-2b injections (Anferon; Tianjin Hualida Biotechnology Co., Ltd., Tianjin, China) were administered subcutaneously for 6 cycles (twice weekly in every 4 weeks cycle) at dosages of 3 million units for patients older than 16 years, and at 3 million units per square meter for those younger than 16 years (capped by 3 million units). Prolonged treatment with IFN-α was permitted at the request of patients.^10^

**Definitions of endpoints**

Non-relapse mortality (NRM) was defined as death without disease progression or relapse. Event-free survival (EFS) events were defined as MRD positivity, relapse, or death from any cause. Leukemia-free survival (LFS) was defined as the survival period with continuous CR. Overall survival (OS) events were defined as death from any cause.

**Variables included in** **univariable and multivariable analysis**

Variables included in univariable analysis were as follows:

Sex, age, *TP53* mutation (yes vs. no), *KMT2A*-rearranged (yes vs. no), complex karyotype (yes vs. no), courses of induction chemotherapy before first CR, courses of consolidation chemotherapy, MRD_con1_ status (positive vs. negative), MRD_con2_ status (positive vs. negative), MRD_bft_ status (positive vs. negative), HCT-CI scores before allo-HSCT, donor type (haploidentical related donor vs. matched sibling donor, unrelated donor vs. matched sibling donor) , donor/recipient gender matching (female to male vs. others), blood group disparity (minor mismatched vs. matched, major mismatched or minor and major mismatched vs. matched), conditioning regimen (TBI-based regimen vs. chemotherapy-based regimen) and graft type (BM + PB vs. PB alone).

Variables that were significant (*P* < 0.1) in univariable analyses were included in the following multivariable analysis.

**Supplementary Fig.1 Diagram of patients enrolled.**

**Supplementary Table 2. Multivariable analysis of risk factors for clinical outcomes after allo-HSCT.**

**Supplementary Table 1 Characteristics of adverse-risk AML patients receiving allo-HSCT**

| **Characteristics** | ***n* = 391 (%)** |
| --- | --- |
| Age |  |
| 16–54 years | 318 (81.3) |
| ≥ 55 years | 73 (18.7) |
| Median age at allo-HSCT, years (range) | 41 (16–69) |
| Gender, *n* (%) |  |
| Male | 220 (56.3) |
| Female | 171 (43.7) |
| *TP53* mutation |  |
| Yes | 35 (9.0) |
| No | 356 (91.0) |
| *KMT2A*-rearranged |  |
| Yes | 77 (19.7) |
| No | 314 (80.3)  9 |
| Complex karyotype |  |
| Yes | 44 (11.3) |
| No | 347 (88.7) |
| Courses of induction chemotherapy before first CR, median (range) | 1 (1–8) |
| Courses of consolidation chemotherapy, median (range) | 2 (0–9) |
| HCT-CI scores before allo-HSCT, *n* (%) |  |
| 0 (low risk) | 276 (70.6) |
| 1–2 (intermediate risk) | 77 (19.7) |
| ≥ 3 (high risk) | 38 (9.7) |
| Donor type, *n* (%) |  |
| Matched sibling donor (MSD) | 76 (19.4) |
| Haploidentical related donor (HID) | 282 (72.1) |
| Unrelated donor (URD) | 33 (8.5) |
| Donor/recipient gender matching, *n* (%) |  |
| Female donor/male recipient combination | 315 (80.6) |
| Others | 76 (19.4) |
| Blood group disparity, *n* (%) |  |
| Matched | 223 (57.0) |
| Minor mismatched | 34 (8.7) |
| Major mismatched or minor and major mismatched | 134 (34.3) |
| Conditioning regimen, *n* (%) |  |
| Chemotherapy-based regimen | 386 (98.7) |
| TBI-based regimen | 5 (1.3) |
| Graft type |  |
| PB | 267 (68.3) |
| BM + PB | 124 (31.7) |
| MNC counts in graft, median (range, ×10^8^/kg) | 9.48 (2.51–23.51) |
| CD34^+^ cell counts in graft, median (range, ×10^6^/kg) | 4.00 (0.38–22.68) |
| Allo-HSCT, allogeneic hematopoietic stem cell transplantation; AML, acute myeloid leukemia; BM, bone marrow; CR, complete remission; HCT-CI, hematopoietic cell transplantation-specific comorbidity index; PB, peripheral blood; TBI, total body irradiation; MNC, mononuclear cell. | |

**Supplementary Table 2 GVHD after allo-HSCT**

|  | Cumulative Incidence% (95% CI) |
| --- | --- |
|  | *n* = 391 |
| 100-day acute GVHD |  |
| Grade I–IV | 35.1% (95% CI: 30.3%–39.8%) |
| Grade III–IV | 5.4% (95% CI: 3.1%–7.6%) |
| 2-year chronic GVHD |  |
| Total | 47.2% (95% CI: 42.1%–52.3%) |
| Severe | 10.2% (95% CI: 6.4%–13.9%) |
| GVHD, graft-versus-host-disease | |

|  | MRD_con1_ positivity  (*n* = 114) | MRD_con1_ negativity  (*n* = 243) | *P*-value |
| --- | --- | --- | --- |
|  | Cumulative Incidence (95% CI) | Cumulative Incidence (%) (95% CI) |  |
| **0-2 years** |  |  |  |
| Relapse | 26.9% (95% CI: 18.1%–35.7%) | 9.4% (95% CI: 5.2%–13.6%) | < 0.001 |
| Event-free survival | 54.4% (95% CI: 45.5%–64.9%) | 78.4% (95% CI: 72.9%–84.3%) | < 0.001 |
| Leukemia free survival | 60.7% (95% CI: 51.7%–71.1%) | 81.5% (95% CI: 76.3%–87.1%) | < 0.001 |
| Overall survival | 74.4% (95% CI: 66.3%–83.3%) | 88.6% (95% CI: 84.4%–93.0%) | < 0.001 |
| Non-relapse mortality | 12.4% (95% CI: 6.0%–18.9%) | 9.1% (95% CI: 5.2%–12.9%) | 0.333 |
| **0-1 year** |  |  |  |
| Relapse | 18.8% (95% CI: 11.5%–26.1%) | 4.3% (95% CI: 1.7%–6.9%) | < 0.001 |
| Event-free survival | 66.2% (95% CI: 58.0%–75.6%) | 84.8% (95% CI: 80.4%–89.5%) | < 0.001 |
| Leukemia free survival | 73.2% (95% CI: 65.4–81.9%) | 89.8% (95% CI: 86.0%–93.8%) | < 0.001 |
| Overall survival | 82.2% (95% CI: 75.4%–89.6%) | 92.4% (95% CI: 89.0%–95.8%) | 0.003 |
| Non-relapse mortality | 8.0% (95% CI: 3.0%–13.1%) | 5.9% (95% CI: 1.7%–6.9%) | 0.430 |
| **1-2 years** |  |  |  |
| Relapse | 11.1% (95% CI: 3.2%–19.1%) | 5.3% (95% CI: 1.6%–9.0%) | 0.395 |
| Event-free survival | 82.1 (95% CI: 72.9%–92.4%) | 92.9% (95% CI: 88.7%–97.4%) | 0.007 |
| Leukemia free survival | 82.9 (95% CI: 74.0%–92.8%) | 91.2% (95% CI: 86.8%–95.9%) | 0.101 |
| Overall survival | 90.5% (95% CI: 84.0%–97.5%) | 95.9% (95% CI: 92.9%–99.0%) | 0.085 |
| Non-relapse mortality | 6.0% (95% CI: 0–54.1%) | 3.5% (95% CI: 0–40.2%) | 0.121 |

**Supplementary Table 3 Clinical outcomes between patients with positive and negative MRD_con1_**

|  | MRD_con1_ positive  (*n* = 45) | | MRD_con1_ negative  (*n* = 59) | | *p*-value |
| --- | --- | --- | --- | --- | --- |
|  | Cumulative Incidence (%) | 95% CI (%) | Cumulative Incidence (%) | 95% CI (%) |  |
| **0-2 years** |  |  |  |  |  |
| Relapse | 42.2 | 26.1–58.3 | 11.0 | 0–22.5 | < 0.001 |
| Event-free survival | 41.3 | 28.6–59.6 | 76.8 | 63.8–92.3 | < 0.001 |
| Leukemia free survival | 46.2 | 32.7–65.2 | 82.2 | 70.1–96.4 | < 0.001 |
| Overall survival | 73.7 | 61.3–88.5 | 93.3 | 86.1–100.0 | 0.004 |
| Non-relapse mortality | 11.7 | 1.9–21.4 | 6.8 | 0–14.5 | 0.274 |
| **0-1 year** |  |  |  |  |  |
| Relapse | 29.8 | 16.0–43.6 | 1.9 | 0–5.7 | <0.001 |
| Event-free survival | 54.7 | 41.8–71.5 | 89.1 | 81.2–97.8 | < 0.001 |
| Leukemia free survival | 63.5 | 50.7–79.5 | 96.3 | 91.4–100.0 | < 0.001 |
| Overall survival | 81.9 | 71.2–94.1 | 98.2 | 94.8–100.0 | 0.004 |
| Non-relapse mortality | 1.8 | 0–5.3 | 6.8 | 0–14.3 | 0.193 |
| **1-2 years** |  |  |  |  |  |
| Relapse | 19.5 | 1.7–37.4 | 7.6 | 0–18.6 | 0.197 |
| Event-free survival | 75.5 | 58.7–96.9 | 88.1 | 75.5–100.0 | 0.098 |
| Leukemia free survival | 72.8 | 56.1–94.5 | 87.1 | 75.4–100.0 | 0.158 |
| Overall survival | 90.0 | 79.8–100.0 | 95.0 | 88.4–100.0 | 0.340 |
| Non-relapse mortality | 7.7 | 0–18.1 | 5.3 | 0–12.6 | 0.539 |

**Supplementary Table 4 Clinical outcomes in patients receiving allo-HSCT directly after the first consolidation chemotherapy**

**Supplementary Table 5. Univariable analysis of clinical outcomes following allo-HSCT**

| **Outcomes** | **HR (95% CI)** | ***P*** |
| --- | --- | --- |
| **Relapse** |  |  |
| Sex |  |  |
| Male | 1 |  |
| Female | 0.48 (0.26–0.88) | 0.018 |
| Age |  |  |
| 16–54 years | 1 |  |
| ≥ 55 years | 0.64 (0.27–1.50) | 0.304 |
| *TP53* mutation |  |  |
| No | 1 |  |
| Yes | 1.30 (0.56–3.04) | 0.546 |
| *KMT2A*-rearranged |  |  |
| No | 1 |  |
| Yes | 1.41 (0.75–2.64) | 0.286 |
| Complex karyotype |  |  |
| No | 1 |  |
| Yes | 1.19 (0.54–2.65) | 0.665 |
| Courses of induction chemotherapy before first CR |  |  |
| 1 | 1 |  |
| > 1 | 3.63 (2.08–6.35) | < 0.001 |
| Courses of consolidation chemotherapy |  |  |
| ≥ 1 | 1 |  |
| 0 | 0.92 (0.33–2.56) | 0.876 |
| MRD_con1_ positivity |  |  |
| No | 1 |  |
| Yes | 3.43 (1.93–6.09) | < 0.001 |
| MRD_con2_ positivity |  |  |
| No | 1 |  |
| Yes | 2.67 (1.25–5.72) | 0.011 |
| MRD_bft_ positivity |  |  |
| No | 1 |  |
| Yes | 3.43 (1.98–5.96) | < 0.001 |
| HCT-CI scores before allo-HSCT |  |  |
| 0 (low risk) | 1 |  |
| 1–2 (intermediate risk) | 0.90 (0.43–1.85) | 0.764 |
| ≥ 3 (high risk) | 0.77 (0.27–2.15) | 0.613 |
| Donor type |  |  |
| Matched sibling donor | 1 |  |
| Haploidentical related donor | 0.95 (0.47–1.92) | 0.885 |
| Unrelated donor | 1.93 (0.73–5.06) | 0.184 |
| Donor/recipient gender matching |  |  |
| Other | 1 |  |
| Female donor/male recipient combination | 1.09 (0.56–2.12) | 0.804 |
| Blood group disparity |  |  |
| Matched | 1 |  |
| Minor mismatched | 1.45 (0.61–3.50) | 0.403 |
| Major mismatched or minor and major mismatched | 0.85 (0.46–1.55) | 0.589 |
| Conditioning regimen |  |  |
| Chemotherapy-based regimen | 1 |  |
| TBI-based regimen | 4.13 (1.00–17.03) | 0.049 |
| Graft type |  |  |
| BM+PB | 1 |  |
| PB alone | 0.47 (0.27–0.82) | 0.007 |
| **Treatment failure as defined by event-free survival** |  |  |
| Sex |  |  |
| Male | 1 |  |
| Female | 0.75 (0.51–1.11) | 0.149 |
| Age |  |  |
| 16–54 years | 1 |  |
| ≥ 55 years | 0.79 (0.47–1.35) | 0.392 |
| *TP53* mutation |  |  |
| No | 1 |  |
| Yes | 0.80 (0.39–1.64) | 0.544 |
| *KMT2A*-rearranged |  |  |
| No | 1 |  |
| Yes | 0.89 (0.55–1.47) | 0.657 |
| Complex karyotype |  |  |
| No | 1 |  |
| Yes | 1.39 (0.82–2.36) | 0.225 |
| Courses of induction chemotherapy before first CR |  |  |
| 1 | 1 |  |
| > 1 | 2.18 (1.50–3.18) | < 0.001 |
| Courses of consolidation chemotherapy |  |  |
| ≥ 1 | 1 |  |
| 0 | 0.92 (0.33–2.56) | 0.876 |
| MRD_con1_ positive |  |  |
| No | 1 |  |
| Yes | 2.65 (1.78–3.93) | < 0.001 |
| MRD_con2_ positive |  |  |
| No | 1 |  |
| Yes | 2.02 (1.21–3.39) | 0.007 |
| MRD_bft_ positive |  |  |
| No | 1 |  |
| Yes | 0.62 (0.51–0.75) | < 0.001 |
| HCT-CI scores before allo-HSCT |  |  |
| 0 (low risk) | 1 |  |
| 1–2 (intermediate risk) | 1.32 (0.84–2.06) | 0.230 |
| ≥ 3 (high risk) | 0.89 (0.45–1.78) | 0.743 |
| Donor type |  |  |
| Matched sibling donor | 1 |  |
| Haploidentical related donor | 1.00 (0.62–1.62) | 1.000 |
| Unrelated donor | 1.45 (0.70–3.01) | 0.318 |
| Donor/recipient gender matching |  |  |
| Other | 1 |  |
| Female donor/male recipient combination | 1.03 (0.64–1.64) | 0.913 |
| Blood group disparity |  |  |
| Matched | 1 |  |
| Minor mismatched | 1.30 (0.69–2.47) | 0.420 |
| Major mismatched or minor and major mismatched | 0.92 (0.61–1.40) | 0.707 |
| Preconditioning regimen |  |  |
| Chemotherapy-based regimen | 1 |  |
| TBI-based regimen | 2.77 (0.88–8.74) | 0.082 |
| Graft type |  |  |
| BM+PB | 1 |  |
| PB alone | 0.45 (0.31–0.66) | < 0.001 |
| **Treatment failure as defined by leukemia-free survival** |  |  |
| Sex |  |  |
| Male | 1 |  |
| Female | 0.67 (0.43–1.04) | 0.075 |
| Age |  |  |
| 16–54 years | 1 |  |
| ≥ 55 years | 0.83 (0.46–1.51) | 0.547 |
| *TP53* mutation |  |  |
| No | 1 |  |
| Yes | 0.73 (0.32–1.67) | 0.452 |
| *KMT2A*-rearranged |  |  |
| No | 1 |  |
| Yes | 1.01 (0.59–1.71) | 0.986 |
| Complex karyotype |  |  |
| No | 1 |  |
| Yes | 1.10 (0.59–2.07) | 0.766 |
| Courses of induction chemotherapy before first CR |  |  |
| 1 | 1 |  |
| > 1 | 2.16 (1.42–3.27) | < 0.001 |
| Courses of consolidation chemotherapy |  |  |
| ≥ 1 | 1 |  |
| 0 | 0.95 (0.44–2.06) | 0.904 |
| MRD_con1_ positive |  |  |
| No | 1 |  |
| Yes | 2.51 (1.62–3.88) | < 0.001 |
| MRD_con2_ positive |  |  |
| No | 1 |  |
| Yes | 1.93 (1.10–3.39) | 0.023 |
| MRD_bft_ positive |  |  |
| No | 1 |  |
| Yes | 0.38 (0.25–0.58) | < 0.001 |
| HCT-CI scores before allo-HSCT |  |  |
| 0 (low risk) | 1 |  |
| 1–2 (intermediate risk) | 1.55 (0.95–2.51) | 0.079 |
| ≥ 3 (high risk) | 1.04 (0.49–2.17) | 0.926 |
| Donor type |  |  |
| Matched sibling donor | 1 |  |
| Haploidentical related donor | 0.87 (0.52–1.45) | 0.586 |
| Unrelated donor | 1.15 (0.50–2.63) | 0.736 |
| Donor/recipient gender matching |  |  |
| Other | 1 |  |
| Female donor/male recipient combination | 1.12 (0.67–1.86) | 0.669 |
| Blood group disparity |  |  |
| Matched | 1 |  |
| Minor mismatched | 1.06 (0.51–2.23) | 0.873 |
| Major mismatched or minor and major mismatched | 0.76 (0.48–1.22) | 0.256 |
| Preconditioning regimen |  |  |
| Chemotherapy-based regimen | 1 |  |
| TBI-based regimen | 3.67 (1.16–11.63) | 0.027 |
| Graft type |  |  |
| BM+PB | 1 |  |
| PB alone | 0.49 (0.32–0.74) | 0.001 |
| **Treatment failure as defined by overall survival** |  |  |
| Sex |  |  |
| Male | 1 |  |
| Female | 0.87 (0.53–1.45) | 0.602 |
| Age |  |  |
| 16–54 years | 1 |  |
| ≥ 55 years | 0.95 (0.48–1.87) | 0.881 |
| *TP53* mutation |  |  |
| No | 1 |  |
| Yes | 0.49 (0.15–1.56) | 0.227 |
| *KMT2A*-rearranged |  |  |
| No | 1 |  |
| Yes | 0.78 (0.40–1.54) | 0.479 |
| Complex karyotype |  |  |
| No | 1 |  |
| Yes | 0.98 (0.35–2.78) | 0.976 |
| Courses of induction chemotherapy before first CR |  |  |
| 1 | 1 |  |
| > 1 | 1.60 (0.97–2.63) | 0.066 |
| Courses of consolidation chemotherapy |  |  |
| ≥ 1 | 1 |  |
| 0 | 1.17 (0.51–2.72) | 0.712 |
| MRD_con1_ positive |  |  |
| No | 1 |  |
| Yes | 2.51 (1.49–4.22) | 0.001 |
| MRD_con2_ positive |  |  |
| No | 1 |  |
| Yes | 1.80 (0.94–3.47) | 0.078 |
| MRD_bft_ positive |  |  |
| No | 1 |  |
| Yes | 0.65 (0.50–0.83) | 0.001 |
| HCT-CI scores before allo-HSCT |  |  |
| 0 (low risk) | 1 |  |
| 1–2 (intermediate risk) | 2.03 (1.18–3.49) | 0.011 |
| ≥ 3 (high risk) | 0.97 (0.38–2.48) | 0.957 |
| Donor type |  |  |
| Matched sibling donor | 1 |  |
| Haploidentical related donor | 0.88 (0.48–1.60) | 0.663 |
| Unrelated donor | 0.71 (0.23–2.14) | 0.538 |
| Donor/recipient gender matching |  |  |
| Other | 1 |  |
| Female donor/male recipient combination | 0.85 (0.44–1.63) | 0.619 |
| Blood group disparity |  |  |
| Matched | 1 |  |
| Minor mismatched | 1.13 (0.48–2.67) | 0.785 |
| Major mismatched or minor and major mismatched | 0.81 (0.47–1.41) | 0.463 |
| Preconditioning regimen |  |  |
| Chemotherapy-based regimen | 1 |  |
| TBI-based regimen | 2.16 (0.53–8.88) | 0.286 |
| Graft type |  |  |
| BM+PB | 1 |  |
| PB alone | 0.42 (0.26–0.70) | 0.001 |
| **Non-relapse mortality** |  |  |
| Sex |  |  |
| Male | 1 |  |
| Female | 1.06 (0.55–2.05) | 0.854 |
| Age |  |  |
| 16–54 years | 1 |  |
| ≥ 55 years | 1.14 (0.50–2.61) | 0.755 |
| *TP53* mutation |  |  |
| No | 1 |  |
| Yes | 0.04 (0.00–6.03) | 0.212 |
| *KMT2A*-rearranged |  |  |
| No | 1 |  |
| Yes | 0.51 (0.18–1.45) | 0.207 |
| Complex karyotype |  |  |
| No | 1 |  |
| Yes | 0.98 (0.35–2.78) | 0.976 |
| Courses of induction chemotherapy before first CR |  |  |
| 1 | 1 |  |
| > 1 | 0.95 (0.47–1.92) | 0.875 |
| Courses of consolidation chemotherapy |  |  |
| 0 | 1 |  |
| ≥ 1 | 0.98 (0.30–3.20) | 0.976 |
| MRD_con1_ positive |  |  |
| No | 1 |  |
| Yes | 1.51 (0.75–3.04) | 0.245 |
| MRD_con2_ positive |  |  |
| No | 1 |  |
| Yes | 1.26 (0.53–3.02) | 0.601 |
| MRD_bft_ positive |  |  |
| No | 1 |  |
| Yes | 0.77 (0.55–1.07) | 0.115 |
| HCT-CI scores before allo-HSCT |  |  |
| 0 (low risk) | 1 |  |
| 1–2 (intermediate risk) | 2.92 (1.45–5.88) | 0.003 |
| ≥ 3 (high risk) | 1.62 (0.55–4.78) | 0.384 |
| Donor type |  |  |
| Matched sibling donor | 1 |  |
| Haploidentical related donor | 0.78 (0.37–1.67) | 0.525 |
| Unrelated donor | 0.29 (0.04–2.27) | 0.237 |
| Donor/recipient gender matching |  |  |
| Other | 1 |  |
| Female donor/male recipient combination | 1.15 (0.52–2.51) | 0.736 |
| Blood group disparity |  |  |
| Matched | 1 |  |
| Minor mismatched | 0.57 (0.14–2.42) | 0.447 |
| Major mismatched or minor and major mismatched | 0.68 (0.32–1.42) | 0.299 |
| Preconditioning regimen |  |  |
| Chemotherapy-based regimen | 1 |  |
| TBI-based regimen | 2.12 (0.29–15.50) | 0.458 |
| Graft type |  |  |
| BM+PB | 1 |  |
| PB alone | 0.51 (0.27–0.98) | 0.043 |
| Allo-HSCT, allogeneic hematopoietic stem cell transplantation; BM, bone marrow; CR, complete remission; HCT-CI, hematopoietic cell transplantation-specific comorbidity index; PB, peripheral blood; MRD_bft_, MRD status before transplantation; MRD_con1,_ MRD status after the first consolidation chemotherapy; MRD_con2,_ MRD status after the second consolidation chemotherapy; TBI, total body irradiation; MNC, mononuclear cell. | | |

**Supplementary Table 6 Clinical outcomes between patients with positive and negative MRD_con2._**

|  | MRD_con2_ positivity  (*n* = 62) | | MRD_con2_ negativity  (*n* = 191) | | *p*-value |
| --- | --- | --- | --- | --- | --- |
|  | Cumulative Incidence (%) | 95% CI (%) | Cumulative Incidence (%) | 95% CI (%) |  |
| **0-2 years** |  |  |  |  |  |
| Relapse | 20.2 | 9.8–30.5 | 8.4 | 4.1–12.7 | 0.007 |
| Event-free survival | 61.3 | 49.9–75.3 | 78.2 | 72.2–84.8 | 0.004 |
| Leukemia free survival | 68.0 | 57.0–81.2 | 81.2 | 75.4–87.5 | 0.015 |
| Overall survival | 76.2 | 65.9–88.0 | 86.1 | 81.0–91.6 | 0.053 |
| Non-relapse mortality | 11.8 | 3.2–19.0 | 10.4 | 5.7–15.0 | 0.676 |
| **0-1 year** |  |  |  |  |  |
| Relapse | 14.5 | 5.7–23.4 | 4.3 | 1.7–7.2 | 0.005 |
| Event-free survival | 70.9 | 60.4–83.2 | 84.0 | 79.0–89.4 | 0.014 |
| Leukemia free survival | 75.7 | 65.8–87.2 | 88.8 | 84.4–93.4 | 0.009 |
| Overall survival | 82.2 | 73.1–92.3 | 90.4 | 86.3–94.7 | 0.070 |
| Non-relapse mortality | 9.7 | 2.3–17.2 | 6.9 | 3.3–10.6 | 0.457 |
| **1-2 years** |  |  |  |  |  |
| Relapse | 7.4 | 0–15.7 | 4.6 | 0.9–8.4 | 0.414 |
| Event-free survival | 86.4 | 75.9–98.4 | 93.1 | 88.5–98.0 | 0.150 |
| Leukemia free survival | 89.8 | 80.8–99.9 | 91.5 | 86.7–96.6 | 0.673 |
| Overall survival | 92.7 | 85.1–100.0 | 95.3 | 91.6–99.1 | 0.471 |
| Non-relapse mortality | 2.7 | 0–8.1 | 3.9 | 0.4–7.3 | 0.730 |

**Supplementary Table 7 Clinical outcomes in patients receiving allo-HSCT directly after the second consolidation chemotherapy**

|  | MRD_con2_ positive  (*n* = 40) | | MRD_con2_ negative  (*n* = 121) | | *p*-value |
| --- | --- | --- | --- | --- | --- |
|  | Cumulative Incidence (%) | 95% CI (%) | Cumulative Incidence (%) | 95% CI (%) |  |
| **0-2 years** |  |  |  |  |  |
| Relapse | 17.6 | 5.6–29.6 | 8.2 | 3.0–13.4 | 0.068 |
| Event-free survival | 62.2 | 48.8–79.3 | 80.1 | 73.1–87.8 | 0.012 |
| Leukemia free survival | 67.3 | 54.2–83.6 | 84.0 | 77.5–91.2 | 0.009 |
| Overall survival | 74.8 | 62.5–89.6 | 88.9 | 83.4–94.8 | 0.020 |
| Non-relapse mortality | 15.1 | 3.8–26.4 | 7.8 | 2.9–12.7 | 0.146 |
| **0-1 year** |  |  |  |  |  |
| Relapse | 15.0 | 3.8–26.2 | 5.0 | 1.1–9.0 | 0.039 |
| Event-free survival | 67.4 | 54.3–0.837 | 84.1 | 77.8–90.9 | 0.016 |
| Leukemia free survival | 69.9 | 57.0–85.7 | 89.9 | 84.7–95.5 | 0.002 |
| Overall survival | 74.8 | 62.5–89.6 | 91.6 | 86.8–96.7 | 0.005 |
| Non-relapse mortality | 15.1 | 3.8–26.4 | 5.0 | 1.1–9.0 | 0.036 |
| **1-2 years** |  |  |  |  |  |
| Relapse | 3.7 | 0–11.0 | 3.5 | 0–7.5 | 0.839 |
| Event-free survival | 92.3 | 82.6–100.0 | 95.3 | 90.8–100.0 | 0.470 |
| Leukemia free survival | 96.3 | 89.4–100.0 | 93.4 | 88.5–98.7 | 0.650 |
| Overall survival | 100.0 | NA | 97.0 | 93.7–100.0 | 0.360 |
| Non-relapse mortality | 0 | NA | 3.0 | 0–6.5 | 0.367 |

**Supplementary Table 8 Clinical outcomes between patients receiving allo-HSCT directly with negative MRD_con1_ and negative MRD_con2_**

|  | MRD_con1_ negativity  (*n* = 59) | | MRD_con2_ negativity  (*n* = 121) | | *p*-value |
| --- | --- | --- | --- | --- | --- |
|  | Cumulative Incidence (%) | 95% CI (%) | Cumulative Incidence (%) | 95% CI (%) |  |
| **0-2 years** |  |  |  |  |  |
| Relapse | 11.0 | 0–22.5 | 8.2 | 3.0–13.4 | 0.988 |
| Event-free survival | 76.8 | 63.8–92.3 | 80.1 | 73.1–87.8 | 0.830 |
| Leukemia free survival | 82.2 | 70.1–96.4 | 84.0 | 77.5–91.2 | 0.670 |
| Overall survival | 93.3 | 86.1–100.0 | 88.9 | 83.4–94.8 | 0.240 |
| Non-relapse mortality | 6.8 | 0–14.5 | 7.8 | 2.9–12.7 | 0.602 |
| **0-1 year** |  |  |  |  |  |
| Relapse | 1.9 | 0–5.7 | 5.0 | 1.1–9.0 | 0.302 |
| Event-free survival | 89.1 | 81.2–97.8 | 84.1 | 77.8–90.9 | 0.350 |
| Leukemia free survival | 96.3 | 91.4–100.0 | 89.9 | 84.7–95.5 | 0.140 |
| Overall survival | 98.2 | 94.8–100.0 | 91.6 | 86.8–96.7 | 0.095 |
| Non-relapse mortality | 6.8 | 0–14.3 | 5.0 | 1.1–9.0 | 0.301 |
| **1-2 years** |  |  |  |  |  |
| Relapse | 7.6 | 0–18.6 | 3.5 | 0–7.5 | 0.602 |
| Event-free survival | 88.1 | 75.5–100.0 | 95.3 | 90.8–99.9 | 0.420 |
| Leukemia free survival | 87.1 | 75.4–100.0 | 93.4 | 88.5–98.7 | 0.510 |
| Overall survival | 95.0 | 88.4–100.0 | 97.0 | 93.7–100.0 | 0.710 |
| Non-relapse mortality | 5.3 | 0–7.5 | 3.0 | 0–6.5 | 0.684 |

|  | MRD_bft_ positivity  (*n* = 124) | | MRD_bft_ negativity  (*n* = 267) | | *p*-value |
| --- | --- | --- | --- | --- | --- |
|  | Cumulative Incidence (%) | 95% CI (%) | Cumulative Incidence (%) | 95% CI (%) |  |
| **0-2 years** |  |  |  |  |  |
| Relapse | 26.4 | 18.1–34.7 | 9.4 | 5.4–13.3 | < 0.001 |
| Event-free survival | 55.2 | 46.8–65.1 | 77.7 | 72.4–83.4 | < 0.001 |
| Leukemia free survival | 61.2 | 52.8–71.0 | 81.6 | 76.6–87.0 | < 0.001 |
| Overall survival | 75.6 | 68.3–83.8 | 87.6 | 83.3–92.0 | < 0.001 |
| Non-relapse mortality | 12.4 | 6.5–18.3 | 9.0 | 5.2–12.8 | 0.167 |
| **0-1 year** |  |  |  |  |  |
| Relapse | 18.6 | 11.7–25.5 | 4.7 | 2.1–7.3 | < 0.001 |
| Event-free survival | 64.0 | 56.0–73.1 | 85.0 | 80.8–89.5 | < 0.001 |
| Leukemia free survival | 70.4 | 62.7–79.0 | 90.8 | 87.3–94.4 | < 0.001 |
| Overall survival | 79.4 | 72.6–87.0 | 93.1 | 90.1–96.2 | < 0.001 |
| Non-relapse mortality | 10.5 | 5.1–15.9 | 5.0 | 2.3–7.7 | 0.194 |
| **1-2 years** |  |  |  |  |  |
| Relapse | 10.6 | 3.0–18.1 | 5.2 | 1.8–8.7 | 0.159 |
| Event-free survival | 85.6 | 77.6–94.3 | 92.0 | 87.9–96.2 | 0.079 |
| Leukemia free survival | 87.0 | 79.2–95.5 | 90.3 | 85.9–94.9 | 0.502 |
| Overall survival | 95.2 | 90.7–99.9 | 94.0 | 90.6–97.6 | 0.868 |
| Non-relapse mortality | 2.5 | 0–5.9 | 4.5 | 1.4–7.5 | 0.600 |

**Supplementary Table 9 Clinical outcomes between patients with positive and negative MRD_bft._**

|  | Patients with maintenance therapy  (n = 60) | | Patients without maintenance therapy  (n = 331) | | *p*-value |
| --- | --- | --- | --- | --- | --- |
|  | Cumulative Incidence (%) | 95% CI (%) | Cumulative Incidence (%) | 95% CI (%) |  |
| **0-2 years** |  |  |  |  |  |
| Event-free survival | 73.8 | 62.8–86.8 | 70.2 | 65.1–75.6 | 0.474 |
| Leukemia free survival | 76.8 | 64.7–91.2 | 74.7 | 69.8–79.9 | 0.524 |
| Overall survival | 94.1 | 87.7–100.0 | 82.1 | 77.8–86.5 | 0.028 |
| **0-1 year** |  |  |  |  |  |
| Event-free survival | 84.5 | 75.7–94.4 | 77.3 | 72.8–82.0 | 0.200 |
| Leukemia free survival | 86.0 | 77.4–94.6 | 84.0 | 80.1–88.1 | 0.630 |
| Overall survival | 96.5 | 91.8–100.0 | 87.4 | 83.8–91.1 | 0.044 |
| **1-2 years** |  |  |  |  |  |
| Event-free survival | 87.4 | 77.6–98.4 | 91.1 | 87.3–95.1 | 0.340 |
| Leukemia free survival | 89.3 | 78.0–100.0 | 89.2 | 85.1–93.6 | 0.710 |
| Overall survival | 97.5 | 92.8–100.0 | 93.9 | 90.8–97.1 | 0.360 |

**Supplementary Table 10 Clinical outcomes in patients with and without maintenance chemotherapy after allo-HSCT**

|  | Patients with maintenance therapy  (*n* = 27) | | Patients without maintenance therapy  (*n* = 97) | | *p*-value |
| --- | --- | --- | --- | --- | --- |
|  | Cumulative Incidence (%) | 95% CI (%) | Cumulative Incidence (%) | 95% CI (%) |  |
| 2-year event-free survival | 66.0 | 50.1–86.8 | 63.3 | 54.4–73.8 | 0.640 |
| 2-year leukemia free survival | 60.6 | 42.0–87.5 | 60.8 | 51.5–71.9 | 0.722 |
| 2-year overall survival | 92.3 | 82.6–100.0 | 71.1 | 62.4–81.0 | 0.034 |

**Supplementary Table 11 Clinical outcomes in MRD_bft_ positive patients with and without maintenance therapy**

**Supplementary Table 12 Clinical outcomes between MRD_bft_ positive patients receiving maintenance therapies and** **MRD_bft_ negative patients without receiving maintenance therapies**

|  | MRD_bft_ positive patients receiving maintenance therapies  (*n* = 27) | | MRD_bft_ negative patients without receiving maintenance therapies  (*n* = 234) | | *p*-value |
| --- | --- | --- | --- | --- | --- |
|  | Cumulative Incidence (%) | 95% CI (%) | Cumulative Incidence (%) | 95% CI (%) |  |
| **0-2 years** |  |  |  |  |  |
| Event-free survival | 57.4 | 41.1–80.3 | 76.6 | 70.9–82.7 | 0.014 |
| Leukemia free survival | 60.6 | 42.0–87.5 | 80.5 | 75.1–86.3 | 0.017 |
| Overall survival | 92.3 | 82.6–100.0 | 86.5 | 81.9–91.4 | 0.550 |
| **0-1 year** |  |  |  |  |  |
| Event-free survival | 66.0 | 50.1–86.8 | 83.0 | 78.3–88.0 | 0.026 |
| Leukemia free survival | 69.2 | 53.5–89.6 | 89.5 | 85.6–93.6 | 0.003 |
| Overall survival | 92.3 | 82.6–100.0 | 92.1 | 88.7–95.7 | 0.980 |
| **1-2 years** |  |  |  |  |  |
| Event-free survival | 87.1 | 71.8–100.0 | 92.7 | 88.4–100.0 | 0.240 |
| Leukemia free survival | 87.5 | 67.3–100.0 | 90.1 | 86.0–94.5 | 0.780 |
| Overall survival | 100.0 | NA | 93.9 | 90.2–97.7 | 0.310 |
|  |  |  |  |  |  |

**Supplementary Table 13 Clinical outcomes between patients receiving maintenance and preemptive therapies after allo-HSCT**

|  | Maintenance therapy  (*n* = 60) | | Preemptive therapy  (*n* = 51) | | *p*-value |
| --- | --- | --- | --- | --- | --- |
|  | Cumulative Incidence (%) | 95% CI (%) | Cumulative Incidence (%) | 95% CI (%) |  |
| 2-year relapse | 20.8 | 7.9–33.8 | 31.4 | 17.9–44.9 | 0.195 |
| 2-year leukemia free survival | 76.8 | 64.7–91.2 | 52.9 | 40.1–69.8 | 0.012 |
| 2-year overall survival | 94.1 | 87.7–100.0 | 71.9 | 59.9–86.3 | 0.004 |
| 2-year non-relapse mortality | 2.3 | 0–6.9 | 15.7 | 4.7–26.6 | 0.022 |
